# Quartz Knapping Strategies in the Howiesons Poort at Sibudu (KwaZulu-Natal, South Africa)

**DOI:** 10.1371/journal.pone.0101534

**Published:** 2014-07-11

**Authors:** Paloma de la Peña, Lyn Wadley

**Affiliations:** Evolutionary Studies Institute, University of the Witwatersrand, Johannesburg, South Africa; University of Oxford, United Kingdom

## Abstract

The variability associated with Sibudu's Howiesons Poort Industry highlights the unpredictable trajectory of technology in the Middle Stone Age. We reach this conclusion through a study of the technology on quartz from one of the Howiesons Poort layers (Grey Sand) from Sibudu rock shelter. Quartz bifacial technology has previously been described at the site, but this new in-depth study of the quartz technology reveals other strategies. First is the recurring employment of bipolar knapping, formerly considered as a defining feature of the Later Stone Age. Secondly, we highlight a laminar technology with emphasis on small quartz bladelets. Bipolar cores are most common, followed by prismatic cores. The knapping strategies in Grey Sand seem to involve systematic recycling and the deliberate production of microliths.

## Introduction

The appearance of microlithic strategies has been one of the main traits used to distinguish Middle (MSA) and Later Stone Age (LSA) technologies [1]–[4]. Moreover, specific knapping methods, such as bipolar knapping (also related to microlithic strategies), have been suggested as a technological marker for recognising the appearance of the LSA [5]–[8]. The original definitions of the terms MSA and LSA [9] were different from the modern ones [8] [10]. Indeed, the new suggestions for distinguishing MSA and LSA have far reaching implications for our interpretations of *Homo sapiens* behavioural development. Notwithstanding that “issues of definition and terminology may seem anachronistic” [3], another opinion is that to dictate definition is to wield cultural power [11]. One way to address behavioural change is to investigate the cognitive implications of certain advances and technological innovations [2] [12]–[14] that appeared during the MSA, but the study of cognition is a developing science so it cannot yet provide definitive behavioural data. Sometimes people in the past made choices mediated by functional need and, on other occasions, non-utilitarian parameters were at play [15] and these will never fit an evolutionary model for technology. In other words, the technological innovations made by early *Homo sapiens* might not always have been required for survival. What remains to be resolved is whether any one behavioural expression is due to a cognitive advance, an arbitrary cultural choice, a functional need, or whether these factors combined simultaneously, a scenario which seems plausible.

Howiesons Poort technology and its functionally-related strategies have sometimes been seen as “precocious” in the development of the MSA [3] [16], with an emphasis on microlithic blanks designed to be used as parts of hunting weapons [17] as well as in other types of tasks [18]. This microlithic character of the Howiesons Poort has led to a variety of interpretations of its place within the development of the MSA. For some time it was considered as a transitional industry to the LSA [3]. The first definition of the Howiesons Poort put the emphasis on the large size of backed tools compared with those in the LSA [19] [20], and only in later research were the truly microlithic strategies (and related small blanks) highlighted [21] [22]. This putatively ‘modern’ character is also found in other expressions of the material culture, for example, complex technology for hafting [13], a varied bone industry [23], and symbolic expressions, such as the ostrich eggshell engravings from Diepkloof [24] and engraved ochre from Klein Kliphuis [25]. While these cultural expressions are now eloquently interpreted as an essential part of the Howiesons Poort, it was initially the lithic assemblages that played the crucial role in characterising the industry, and many aspects of its technology remain to be defined and their significance interpreted.

In this paper we present the technology on quartz of one of the Howieson Poort layers from Sibudu rock shelter: Grey Sand (GS). We focus on quartz because the management of this mineral is quite different from that of the rock types knapped at Sibudu (hornfels and dolerite) [26] [27]. Clarkson [28] has demonstrated that there is regional variation in core reduction in the Howiesons Poort, but we show here that variation can also occur at a single site when rock types are analysed separately. Blade production from prismatic cores was common for hornfels and dolerite. Moreover, the previously defined ‘Klasies Howiesons Poort cores’ [29] have also been found on hornfels in Sibudu's layer GS. Furthermore, blade and bladelet production on hornfels and dolerite demonstrate bimodal size distribution because prismatic cores produce large blanks, and the cores on flakes produce small blanks. Bipolar knapping was only used occasionally for these two rock types [27].

Coming back to the quartz it must be stressed that, apart from quartz bifacial technology [30], there is recurrent employment of bipolar knapping within Howiesons Poort technology. This seems to be a systematic recycling phenomenon. Also, we highlight a laminar technology, with the emphasis on small elongated quartz blanks, usually labelled bladelets. Both knapping methods are observed as genuine microlithic strategies in later contexts, particularly in the LSA. Our purpose here is to describe and demonstrate the systematic character of these technological solutions during Sibudu's Howiesons Poort, and put them into perspective within the MSA sequence of South Africa. The cognitive implications of the advances represented by backed tools have already been pointed out, both in the Howiesons Poort context and in much more ancient archaeological contexts [1] [2] [31] [32]. We shall explain that Sibudu's Howiesons Poort quartz technology is not an exception, but another example of variability within the MSA [28] [33]–[35]. If it does not appear in other so-called Howiesons Poort assemblages or later in post-Howiesons Poort assemblages, this may be because different cultural or functional choices came into play.

### Sibudu and the Howiesons Poort layer, Grey Sand (GS)

Sibudu is located approximately 40 km north of Durban, and about 15 km inland of the Indian Ocean, on a steep cliff overlooking the uThongathi River (29.522627°S, 31.085895°E). The shelter is 55 m long and 18 m in breadth and has a long occupation sequence with several layers and features corresponding to the pre-Still Bay, Still Bay, Howiesons Poort, post-Howiesons Poort, late MSA, final MSA and Iron Age [36].

**Figure 1:**
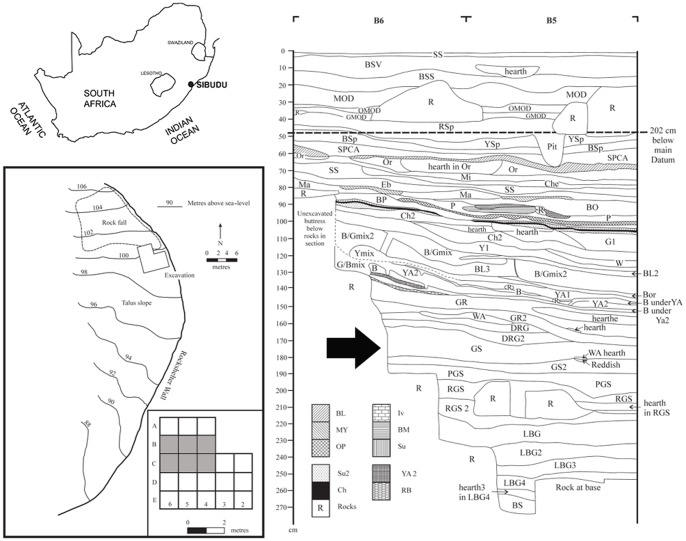
The location of Sibudu, the excavation grid and layer Grey Sand. Top left: location of Sibudu Cave in Southern Africa: 29.522627°S, 31.085895°E. This schematic map was made on the basis of a topographic map of Southern Africa, source: Maps at the CIA (public domain): https://www.cia.gov/library/publications/the-world-factbook/index.html. Bottom left: map of Sibudu with the squares analyzed in this paper highlighted in grey. Right: stratigraphy of the north wall of squares B6 and B5. The Grey Sand (GS) layer has been highlighted with an arrow.

Howiesons Poort occupations reported here come from six square metres (squares B4, B5, B6, C4, C5 and C6) of Wadley's excavations in the deep sounding. The layers associated with the Howiesons Poort are (from the base to the top): Pinkish Grey Sand (PGS), Grey Sand (GS, GS2 and GS3), Dark Reddish Grey (DRG) and Grey Rocky (GR and GR2) (Figure 1).

The stratigraphy is clear, combustion features are discernible [37], and micromorphology implies that most Sibudu layers have stratigraphic integrity [38], although rock fall between the oldest Howiesons Poort layer, PGS, and the underlying Still Bay layer, Reddish Grey Sand (RGS), has caused some disturbance. Earlier rock fall also disrupted pre-Still Bay layers.

Layer Grey Sand (GS) discussed here has an average thickness of 20 cm, and its field description is silty sand with ash and many small rock spalls, and a Munsell colour reading of 5 YR 5/1, grey. GS2 and GS3 are spits to divide GS artificially for excavation purposes, thus GS is about 10 cm deep and GS2 and GS3 are, together, also about 10 cm thick. GS2 has an age estimate of 63.8 kyr obtained from single grain optically stimulated luminescence [39] [40]. The choice of this layer for the detailed analysis of the quartz technology stems from a preliminary analysis conducted on the Howiesons Poort assemblages during which a larger sample of cores and quartz products was identified in GS compared to the other Howiesons Poort layers in Sibudu. In other words, the quartz material in this layer possessed, *a priori*, some ideal conditions for reconstructing the complete quartz *chaîne opératoire*.

### The rocks knapped at Sibudu

The uThongathi River below Sibudu is a source of weathered and river-rolled dolerite and quartzite as well as of small quartz pebbles. This river does not contain hornfels pebbles [26]. The rounded cortex on a number of Sibudu cores and flakes implies a waterborne origin for some dolerite brought to the site, but cores and flakes made from tabular dolerite pieces are also present. Abundant igneous dolerite near Sibudu derives from intrusive Jurassic volcanism, mostly as sills, although a true dolerite dyke lies close to the rock shelter [41] and this seems likely to have been the source of much of the dolerite at the site. Dolerite sills in the area include fine-grained ones like the Mhlasini sill and coarse-grained ones like the Verulam sill [41]. Petrographic analysis of a thin section of fine-grained dolerite showed that the minerals include 45% clinopyroxene, 44.5% plagioclase and small percentages of quartz, limonite and goethite [36]. Unfortunately, dolerite is chemically similar across large regions of South Africa and cannot be distinguished, although the Effingham sills in KwaZulu-Natal have higher silica contents (in excess of 63 wt%) than most of the others [41].

Where dolerite intrudes into shale, there are bands of metamorphic hornfels. Differing temperatures occur in the zone of thermal metamorphism where a dolerite intrusion occurs. Consequently, there are different grades of both hornfels and dolerite. Hornfels is hard to find in the Sibudu area, but one source is near Verulam, about 15 km from Sibudu [42]. This hornfels is poorly metamorphosed, and occurs as thin slabs, only a few centimetres thick. XRF elemental analysis of a piece of hornfels from Sibudu demonstrated its high silica content (63.8%) [43].

Dolerite grain sizes are significantly larger than those of hornfels, and dolerite has a rougher surface than hornfels because the platy plagioclase grains within it are larger than the quartz grains in hornfels [26]. The greater the surface roughness of rock types, the less the wear on tool edges, so roughness is a decisive attribute influencing the rate of wear on a tool. Cochrane [42] found that far more hornfels retouched tools are broken than dolerite retouched tools; dolerite tools seem to hold their edges better than those made on hornfels. Fracture toughness (brittleness) is another important attribute because increased toughness makes knapping difficult. A low energy Charpy pendulum impact testing machine was used for measuring (in joules/mm2) the toughness of dolerite and hornfels [26]. The greater toughness of dolerite over hornfels is part of the reason why dolerite is so much more difficult to knap than hornfels.

Quartz can be divided into two broad categories: crystalline quartz, commonly called macrocrystalline quartz, and the dense and compact forms, which usually are named cryptocrystalline or microcrystalline. The differences between these two broad categories are simply a consequence of the way they form. Macrocrystalline quartz grows by adding molecules to the crystal's surface, whereas cryptocrystalline forms come from colloidal watery solutions of silica. Both varieties appear at Sibudu. However, cryptocrystalline material, such as chalcedony, is extremely rare in Sibudu's Howieson Poort layers. On the contrary, crystalline quartz is very abundant. Within the crystalline quartz assemblage at Sibudu we can distinguish three main categories: vein quartz (milky), crystal quartz and rock quartz (glassy), which is particularly prolific in GS. Crystal quartz is also called hyaline quartz; this is a crystalline form occurring in different geological contexts, including geodes and hydrothermal vein formations. Vein and crystal quartz, which are present in small quantities in GS, were the subject of previous publications [21] [44]. During the technological analyses we did not make the distinction between these last categories, owing to the fact that colour and transparency are highly subjective parameters. Quartz is generally 100% silica, or silicon dioxide (SiO_2_), so it is not granular except when it occurs as an impure form with inclusions of sand, clay or various minerals [45]. It is hard (7 on Moh's scale) so it produces long-lasting, sharp tool edges. Its disadvantage is that it contains faults and routinely shatters, often causing tools to break during knapping [46]. As mentioned before, most of the crystalline quartz pieces found at Sibudu seem to have been collected from the uThongathi River in the form of small pebbles, with a 6 cm average length. The quartz may have originated in the granite outcrops north of Sibudu.

**Figure 2:**
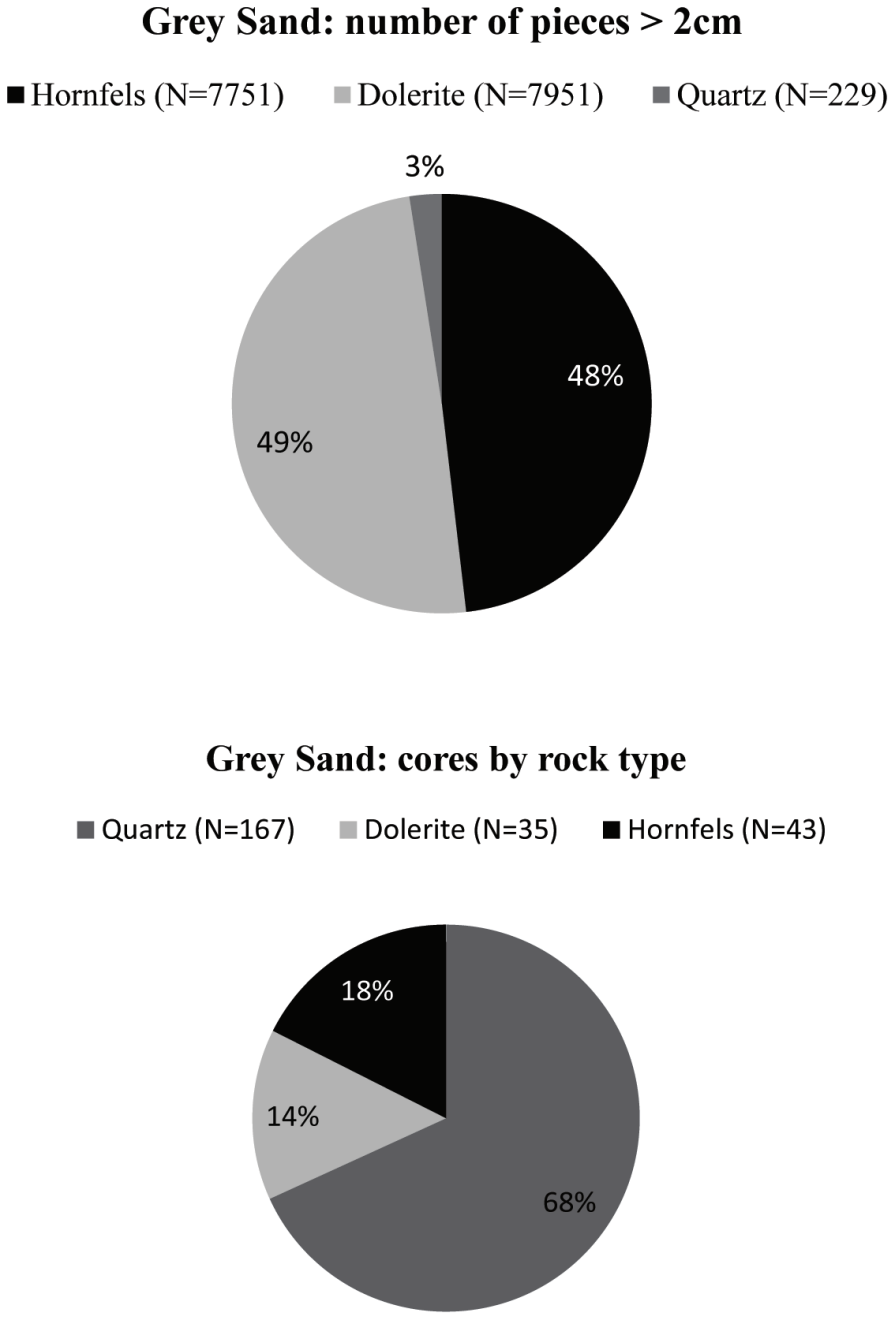
Sibudu, layer GS. On top, percentage of pieces over 2

Hornfels and dolerite pieces larger than 2 cm have a 48 and 49% representation in GS; but quartz does not even reach 3% in squares B4 and B5, 2]. However, this proportion inverts completely if the representation of cores by rock type is taken into account (Figure 2). In this case the percentage of quartz cores is overwhelmingly higher than that of hornfels and dolerite cores. This means that quartz was intensely exploited, but not for pieces larger than 2 cm (Figure 2). Indeed, a total of 15564 pieces of quartz has been analysed, but more than 80% are chips (Table 1). Among the *debitage*, platform flakes are the most represented blank, then bipolar blanks and blade/bladelets (Table 2).

**Table 1:** Frequencies, percentages and weights of the main technological categories in quartz.

Total amount quartz			
Categories	Pieces (n)	%	Weight
Non retouched pieces (>1 cm)	1960	12.59	1151.6
Used pieces	267	1.71	107.23
Chips	12948	83.19	1183.68
Retouched pieces	216	1.38	132.05
Cores	173	1.11	255.51
**Total**	**15564**	**100**	**2830.07**

**Table 2:** Main categories of non retouched pieces in quartz.

Pieces>1 cm (Non retouched pieces)	N	%
Platform flake	588	30
Bipolar flake	379	19.34
Blade/Bladelet	221	11.28
Fragment without platform	585	29.85
Chunk	187	9.54
**Total**	**1960**	**100**

## Methodology

The excavation of Sibudu was conducted with a permit obtained from Amafa i KwaZulu-Natali, the Heritage Agency based in Pietermaritzburg, South Africa. No ethics clearance or permit is required to study the lithic artefacts from Sibudu.

For the general industrial study we followed the *chaîne opératoire* approach [47]–[49]. In fact, the main objective of this paper is to understand the entire cycle of collection, production, use and recycling of quartz in Sibudu's Howiesons Poort. However, quartz is a rock that does not produce the same type of blanks as other siliceous rocks, such as flint or fine quartzite, thus, we also followed specialized methods focused exclusively on quartz.

**Figure 3:**
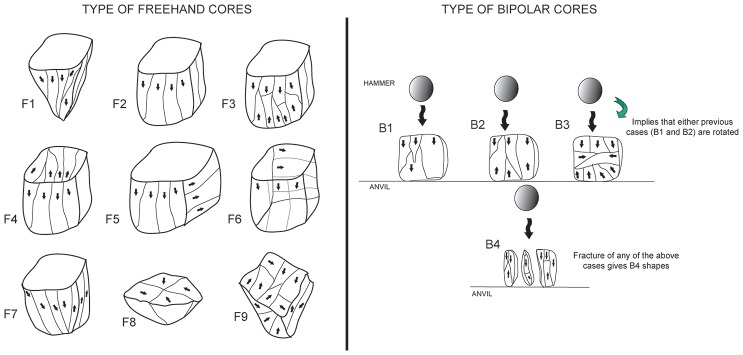
Freehand and bipolar cores from Sibudu's layer GS. Left: types of freehand cores in GS. F1 conical core; F2, F3, F4, F5, F6 and F7 various types of prismatic cores (with different directions of removals); F8 centripetal core; F9 multifacial core. Right: different types of bipolar cores considered in this study, with different directions of removals. B1. Unidirectional. B2. Bidirectional. B3 is a result of rotation. B4 is typical fracture accidents of bipolar knapping.

Sibudu's cores have been studied using the attributes presented in previous quartz studies such as the ones of: Callahan [50], Knutsson [51], Driscoll [52], and Díez-Martín [53]. We have added other attributes. The selected attributes take into account not only the general size of the pieces, but measurements of the knapping surface and specific characteristics such as fissures, blunting, etc., that are typical of bipolar knapping, even though freehand knapping of quartz may also sometimes produce such attributes [52]. The main attributes of the cores are recorded in Table 3; as can be seen there is a mix of quantitative and qualitative variables. Moreover, we propose two different specific classifications for bipolar cores and freehand cores (Figure 3). The classification of cores has been made with consideration for the direction of the removals (for the freehand group) and for the typical reduction cycle of bipolar knapping (for bipolar cores), which has been the subject of previous research [55] [56].

**Table 3:** Variables taken into account for the quartz core study.

Variables taken into account for the core quartz study	Type of blank
	Presence of cortex
	Length, Breadth, Thickness and Weight
	Volumetric shape
	Number of striking platforms
	Orientation of striking platforms
	Length and Breadth of striking platforms (in case of several the larger)
	Type of preparation of the striking platform
	Length and Breadth of knapping surface
	Geometric shape of knapping surface
	Orientation of last negatives
	Length and Breadth of last negative
	Presence of conchoidal negatives (Yes/No)
	Presence of fissuration on overhang or striking platform (Yes/No)
	Presence of bluntness on overhang or striking platform (Yes/No)
	Freehand or Bipolar cores
	Type of cores (See Figure 2)
	Recycled core (from freehand to bipolar)
	Comments/Observations

All the quartz debitage, meaning all the by-products of knapping, excluding retouch, has been classified in the following categories: platform flake, bipolar flake, blade/bladelet, fragment without platform, chunk and chip. A platform flake is a flake that can be oriented, and on which all attributes such as platform, dorsal and ventral faces are visible. In this category we have also included broken proximal flakes [52]. A bipolar flake is a flake with linear or broken platform, and fissuration of its proximal dorsal part, absence of bulb and rectilinear profile. It is what Cotterel and Kamminga [57] call a compression flake. A bladelet is an elongated by-product with parallel ridges and breadth less than 12 mm. A flake fragment without platform retains the ventral or dorsal face. A chunk is a rock fragment where it is not possible to recognize knapping attributes such as platform or bulb, and the piece is clearly not a flake. A chip is a piece less than 10 mm. Small bladelet fragments are excluded from this category because they were analysed with the bladelet fragments greater than 10 mm. All the pieces have been both counted and weighed.

All the pieces greater than 10 mm were examined under a Leica microscope A60 (magnifications from x5–x30) in search of macroscopic use-wear, which is a first sign of use or hafting [51]. In so doing, we have also recorded all the pieces that have signs of utilization; they were counted and weighed separately.

The study of the blanks of the retouched material was more detailed than the study of unretouched pieces, and the classes are listed in Table 4. Again, a mixture of quantitative and qualitative variables (mainly technological) has been applied.

**Table 4:** Variables taken into account for the blanks of the retouched quartz pieces.

Variables taken into account for the blanks of the retouched quartz pieces	Type of blank
	Length, Breadth, Thickness and Weight
	Shape
	Complete or Incomplete
	Break type (following 51)
	Fragment class (following 51)
	Type of platform (Platform flake, Bipolar flake, Blade/bladelet, Fragment without platform, chunk and chip)
	Length and Breadth of platform
	Presence of fissuration on the platform (Yes/No)
	Presence of bluntness on the platform (Yes/No)
	Presence of bulb on the platform (Yes/No)
	Type of dorsal removals (centripetal, unidirectional, bidirectional, multiple)
	Presence of cortex
	Position of retouch
	Type of retouch
	Delineation of the edge where there is retouch
	Location of retouch (following the same fragment class divided by 51)

Even though the emphasis of this paper is on the technology, we have also used broad typological categories, following previous southern African classifications [58] [59], see Table 5, in order to compare Sibudu's GS assemblage with other Howiesons Poort assemblages. During the analysis, we found some morphotypes that have not been recorded in previous studies, such as very small, carefully retouched notches (single and double, see below), which, because of their size and morphology, do not resemble the strangulated pieces and notches reported from other Howiesons Poort sites. However, this will be explained in more detail below.

**Table 5:** Classification of retouched pieces.

General Morphotypes for quartz retouched pieces	Borer
	End-Scraper
	Marginal retouched bladelet
	Marginal retouched bipolar blank
	Retouched flake
	Retouched bipolar flake
	Double Notch
	Single notch
	Denticulated flake
	Denticulated blade
	Bifacial piece
	Ind. denticulated piece
	Diverse (piece with some retouch not completed)
	Ind. Backed (piece not completed with some abrupt retouch)
	Backed blade/bladelet
	Double truncation
	Single truncation
	Segment
	Triangle
	Trapeze

Typometrical and statistical analyses to compare freehand and bipolar cores were performed with the free software PAST: box-plots of variables, normality statistical analysis (Shapiro-Wilk) and additional tests in order to gain support for the technological arguments (U Mann-Whitney and t-test).

## Results

### Quartz lithic technology: Main knapping methods

The main knapping methods in quartz are: flaking in order to obtain blanks to be transformed into bifacial pieces [30], laminar methods in order to obtain blade/bladelets, and bipolar knapping as a recycling strategy of the other two knapping methods.

Beginning with the bifacial technology, it must be explained that some of the unfinished bifacial pieces are made on flakes typical of a discoidal knapping method. However, an expedient method is more likely to have been used than discoidal knapping because among the *debitage* there are no typical discoidal by-products. The absence of these ‘technologically discoidal’ flakes might be also because most of the quartz material has been highly recycled (see below). The brevity and simplicity of the point reduction sequence may partly reflect the high risk of knapping accidents with quartz [46] [50]–[52]. Such a ‘minimum effort strategy’ involves selecting a uniformly thick flake, of maximum thickness required for the completed biface, then shaping and thinning its edges with few flake removals. We hypothesise four phases: selection of the blank, minimal thinning, shaping and final shaping [30].

**Figure 4:**
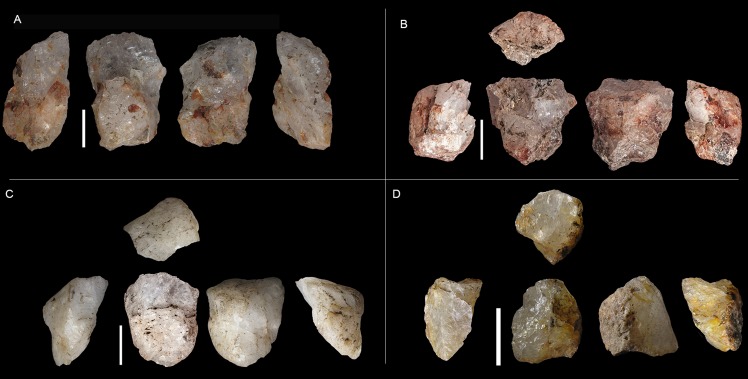
Sibudu, layer GS: examples of prismatic freehand cores. A and B, prismatic cores (Type F2). C and D, pyramidal-unipolar cores (Type F1).

Blade and bladelet production is mainly from prismatic freehand cores (Table 6). These cores come from small river pebbles and have either modest preparation or none at all. A simple flake was usually removed to prepare the striking platform; meanwhile for the knapping surface, the longest side of the core was generally chosen. For freehand cores, unifacial (36.8%) and opposed (47.4%) removals are dominant. The most typical morphotypes of this classification (Figures 3 and 4) are pyramidal-unipolar cores (F1) and opposed platform prismatic cores (F7). Most of the laminar freehand cores found are completely exhausted in terms of knapping and have plenty of negative scars from accidental step and hinge fractures.

**Table 6:** Number and percentage of different core types in Sibudu's layer.

General categories	Types	N	%
Freehand	F0 (tested core, less than 3 negatives)	1	4
	F1	6	24
	F2	4	16
	F3	4	16
	F5	1	4
	F6	1	4
	F7	5	20
	F8	2	8
	F9	1	4
	**Total**	**25**	100
Bipolar	B1	3	2.3
	B2	61	47
	B3	7	5.3
	B4	60	46
	**Total**	**131**	100
Others	Freehand recycled in bipolar	3	27
	Indeterminated (core fragments and doubts)	8	73
	Total	11	100
Total (all type of cores)		167	

**Figure 5:**
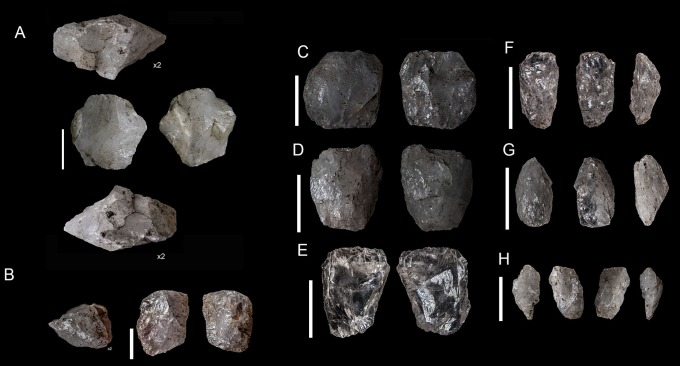
Sibudu, layer GS: examples of bipolar cores. A, B, C, D and E, B2 of our classification. F, G and H, B4 of our classification. In piece A the two opposed edges have been magnified, note their bluntness.

Bipolar knapping, in contrast to freehand knapping, is defined as a method in which a rock is placed on an anvil and held with the bare hand. The rock is hit on the top, causing blanks to be removed from here and also from the edge which is in direct contact with the anvil [60]. Therefore, bipolar cores have two opposed striking platforms (one from direct percussion and the other one from contact with the anvil). Usually, bipolar cores present quadrangular or rectangular shapes. Both the striking platform and the edge in contact with the anvil are rectilinear with much evidence for blunting and splintering, and a striking platform-surface with a knapping angle close to 90 degrees. Nonetheless, when small flakes or chunks are recycled through bipolar knapping, the angle between the platform and face of the piece can be very sharp (personal experimental observation). The most typical bipolar-core-types (Figures 3 and 5) in GS are B2 and B4. However, B4 should be interpreted as a B2 core split in two (see Figure 3), in other words, as an accident from this type of knapping [52].

**Figure 6:**
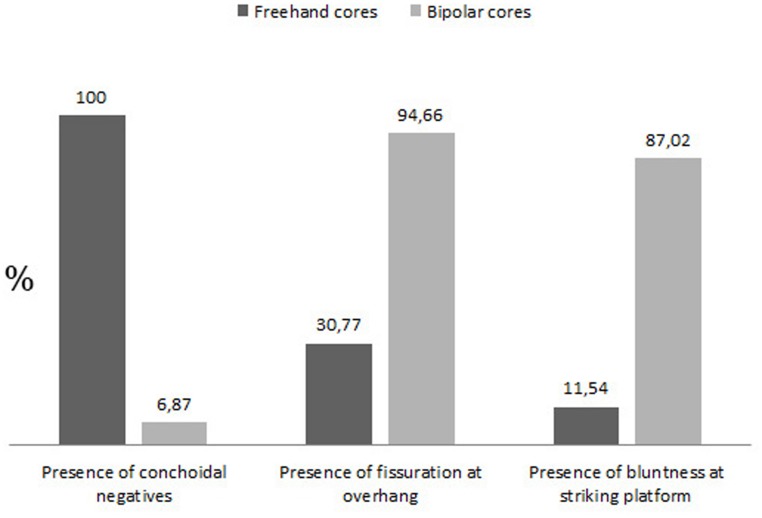
Sibudu, layer GS: Percentage of conchoidal negatives, fissuration and bluntness of freehand and bipolar cores.

**Figure 7:**
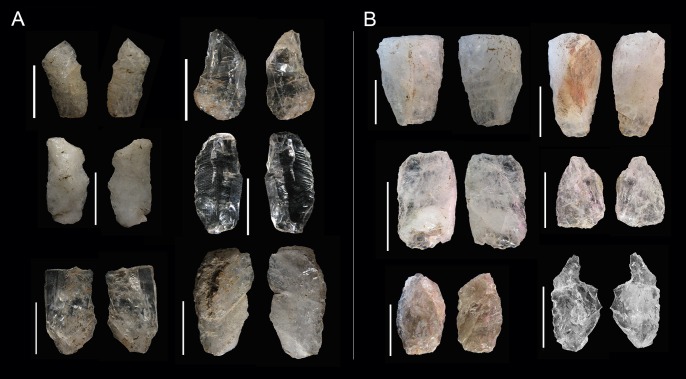
A. Examples of bladelets from GS. B Examples of bipolar blanks from GS.

The distinction between the two main categories of cores (freehand and bipolar) has been recognised during experimental work which has allowed observations of different qualitative characteristics [46] [50]–[54], for example, conchoidal negatives are present abundantly on freehand cores, and bluntness and fissuring are visible on the striking platforms of bipolar cores. As can be seen in Figure 6 the presence of conchoidal scars is uncommon in bipolar cores, whereas fissuring and bluntness are abundant. Fine grained rock types (such as flint) produce characteristic bipolar *debitage* [55], which is not always the case for quartz where freehand and bipolar by-products are quite difficult to distinguish. It is normally accepted that the distinction between the two knapping methods is easier to make on cores than by-products (examples of bladelets from freehand cores and bipolar blanks from bipolar knapping can be seen in Figure 7), especially when dealing with quartz [53] [61].

**Figure 8:**
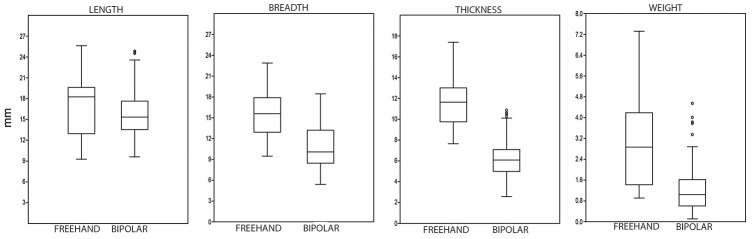
Box-plots of length, breadth, thickness and weight of freehand and bipolar cores from layer GS.

**Figure 9:**
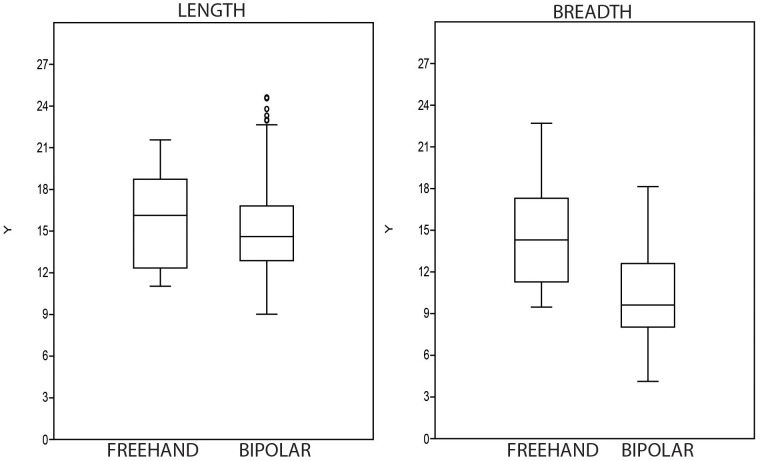
Length and breadth of knapping surface in freehand and bipolar cores from layer GS.

The small size of bipolar cores in the GS sample is particularly striking. In this regard, compare univariate data from freehand and bipolar core measurements (Table 7 and Figure 8). The same situation applies to the knapping surfaces (Figure 9), which are very similar to the maximum size of the bipolar cores; this is because bipolar knapping extractions tend to cover the entire surface of the core. It seems that cores were seldom rotated, and in this regard, note that only 5.3% of cores are the B3 type (rotated bipolar cores). Nonetheless, the possibility that rotation and subsequent striking have obliterated the evidence of the earlier orientation should be also considered.

**Table 7:** Univariate data from freehand and bipolar cores.

	Length		Breadth		Thickness		Weight	
	Freehand	Bipolar	Freehand	Bipolar	Freehand	Bipolar	Freehand	Bipolar
N	19	130	21	129	21	131	23	130
Minimal value	9.27	9.61	9.47	5.4	7.64	2.57	0.91	0.11
Maximal value	25.63	24.83	22.88	17.12	17.4	10.87	7.32	4.55
Mean	17.59	15.79	15.91	10.65	11.68	6.23	3.07	1.22
Std. error	1.01	0.295	0.87	0.26	0.59	0.16	0.37	0.07
Variance	19.59	11.33	16.1	8.99	7.29	3.18	3.18	0.67
Standard deviation	4.43	3.37	4.01	2.99	2.7	1.78	1.78	0.81
Median	18.25	15.33	15.6	10.08	11.64	6.08	2.87	1.04
Skewness	−0.03	0.74	0.2	0.34	0.49	0.46	0.89	1.54
Kurtosis	−0.36	0.29	−0.965	−0.8	−0.13	−0.09	0.16	3.03
Geom. mean	17.03	15.46	15.42	10.23	11.39	5.97	2.61	0.99
Coeff. var	25.15	21.31	25.21	28.16	23.13	28.63	58.12	66.78

### Statistical distinction between freehand and bipolar cores

**Figure 10:**
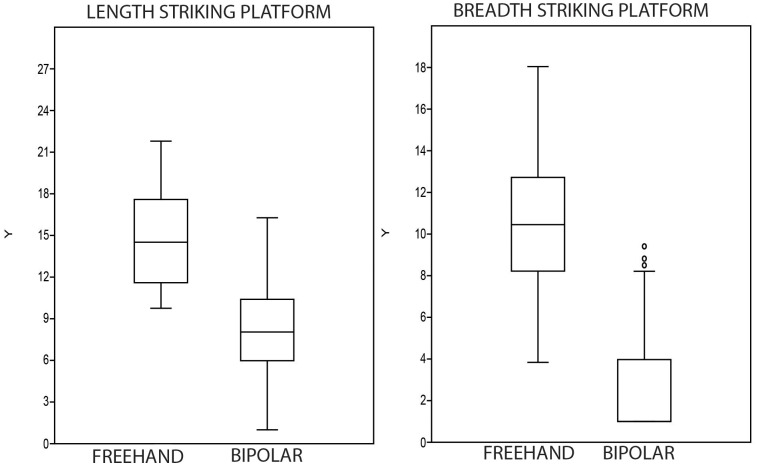
Length and breadth of striking platforms on freehand and bipolar cores from layer GS.

**Figure 11:**
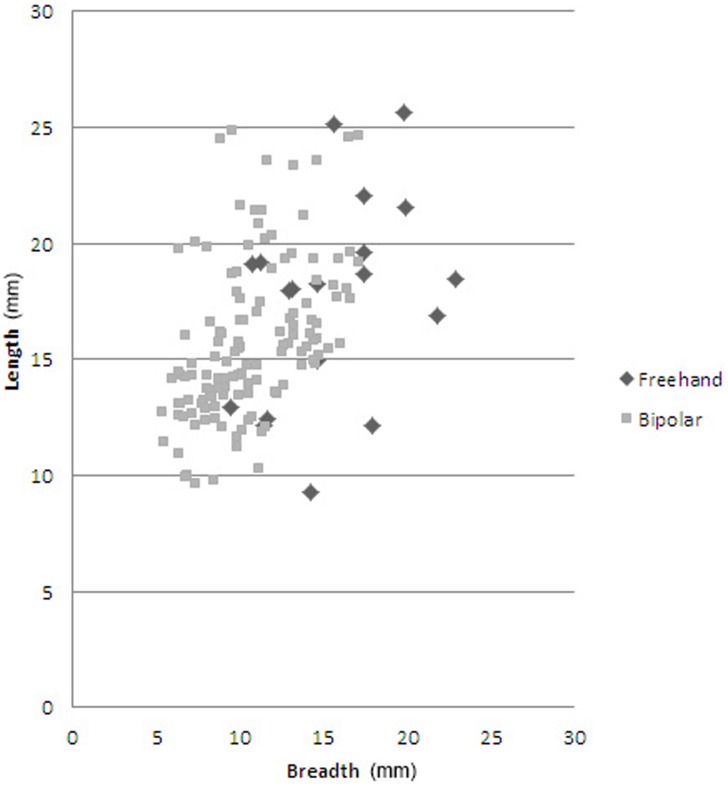
Scatterplot of the lengthening index of freehand and bipolar cores from layer GS.

**Figure 12:**
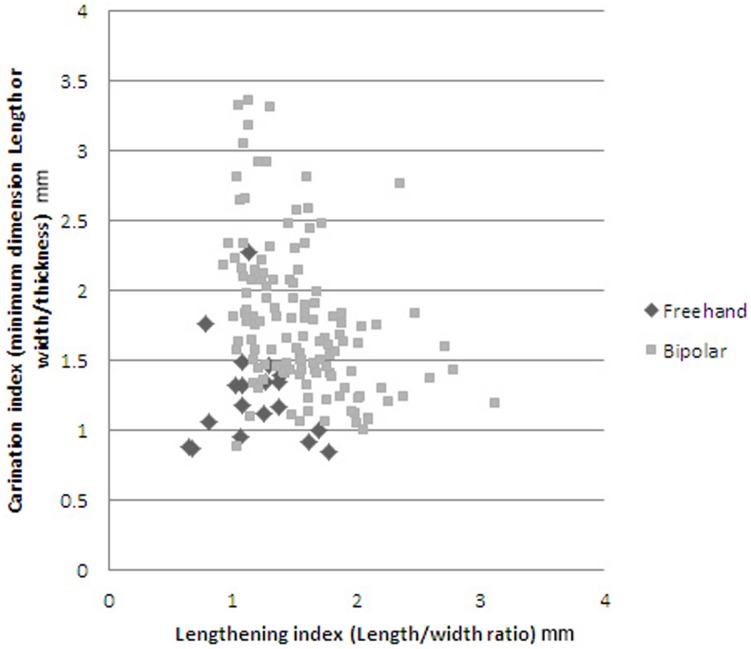
Scatterplot of lengthening index and carination index of freehand and bipolar cores from layer GS.

Apart from the different qualitative characteristics of freehand and bipolar cores (cf. Figures 4 and 5), there are also differences in typometric distributions. If the size distribution of the length, breadth, thickness and weight of bipolar cores and freehand cores is compared, there is a slight difference, and bipolar cores seem systematically smaller (Table 7). This phenomenon is verifiable taking into account the maximum measurements of cores (Figures 8) and also their striking platforms (Figure 10) and knapping surfaces (Figure 9). Besides, the box-plots (Figures 8, 9 and 10) also show that freehand cores are systematically more variable, because the central box (in 75% of the cases) is bigger than that of the bipolar cores. We have also used the lengthening index (LI, the length/width ratio) and carination index (CI, the ratio of the minimum dimension [either length or width] and thickness) in order to compare these two types of cores [53]. The LI differences are not clear enough to show a typometrical distinction between the cores (Figure 11). This may be because, systematically, bipolar cores have been guided by their longest axis, generating an apparent overlap with freehand cores. However, the CI shows a substantial difference between the two categories (Figure 12). Furthermore, the LI and CI of bipolar cores are negatively correlated: the bigger the LI, the smaller the CI. These typometrical insights suggest that the distinction that we have made between freehand and bipolar cores is appropriate.

The difference between these two types of cores is also recognisable statistically. In order to compare freehand and bipolar cores, a normality test was performed as a first step. The Shapiro-Wilk test demonstrates that length, breadth and weight of bipolar cores and weight of freehand cores do not have normal distributions (Table 8). To support the interpretation of a significant difference between freehand and bipolar cores, we performed a U Mann-Whitney test which demonstrated that the two independent variables (core types) are statistically significant for breadth and weight but not for length (Table 9). A T-test was performed in order to compare thickness data (because the data of both core types are normally distributed) which also showed that they are statistically significantly different (Table 9).

**Table 8:** Shapiro-Wilk normality tests. Cases which are not normal have been highlighted with an asterisk.

	Shapiro-Wilk tests							
	Length		Breadth		Thickness		Weight	
	Freehand	Bipolar	Freehand	Bipolar	Freehand	Bipolar	Freehand	Bipolar
W	0.9568	0.9552	0.9555	0.9645	0.9621	0.9757	0.9126	0.8748
p(normal)	0.512	0.00031*	0.4303	0.001955*	0.5588	0.1939	0.04631*	5.96E-09*

**Table 9:** Results of the statistical tests.

Statistical test	U Mann-Whitney test	T-test
Variable	p (same)	
Length	0.07097	
Breadth	2.721E-07*	
Thickness		0.0057*
Weight	5.97E-08*	

*Denotes significant difference.

A plausible hypothesis for the representation of the cores (Table 6) is that many freehand cores were transformed into bipolar cores, because the representation of bipolar cores is more abundant, and there are some examples of bipolar cores with characteristics of freehand cores evident from a previous knapping cycle, as if these were recycled through anvil percussion. Maybe when freehand knapping was no longer possible, because of the small size of the freehand core, the knappers systematically switched to bipolar knapping as in other archaeological contexts [62]. This idea is also supported by the percentage of cortex on cores: as can be seen in Table 10, freehand cores have, in general terms, a larger proportion of cortex. This helps to explain the existence of a greater number of bipolar cores than platform cores; the latter were recycled as bipolar cores.

**Table 10:** Cortex frequency on freehand and bipolar cores.

	Freehand		Bipolar	
Cortex representation	N	%	N	%
0% cortex	14	54	123	92
<50% cortex	7	27	10	8
>50% cortex	5	19	0	0
90–100% cortex	0	0	0	0
∑	26	100	133	100

### Retouched pieces and used quartz pieces

Usually retouched pieces are considered one of the main objectives of knapping activities. This ‘finalist’ concept of industries can give a false picture of the real objectives of knapping in the past. Keeping this idea in mind, we note the high frequency of pieces without retouch that exhibit macro-traces that are probably from use. Indeed, in the total assemblage, they occur more commonly than retouched pieces (Table 1). This is the reason we present them here together with the retouched pieces and other formal tools. Yet, in the absence of a specialised use-trace analysis, we cannot entirely rule out the possibility of a taphonomic explanation for the macro-traces.

**Figure 13:**
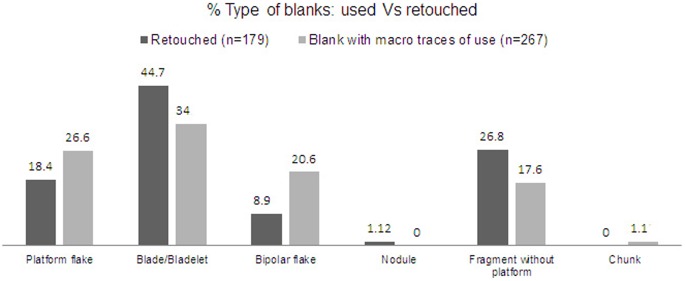
Comparison of percentages of blank types subsequently retouched or used in layer GS.

It is also technologically interesting to compare the percentages of used and retouched blanks. As can be seen in Figure 13, blade/bladelets are the most common blanks for retouched pieces, whereas bipolar flakes are in the minority. However, bipolar flakes have a notable representation among used pieces. This might signify that most bipolar knapping products were used without any kind of retouch. Also, the relatively high proportion of ‘fragments without platforms’ used as blanks for retouched tools may be partly due to platforms being obscured by retouch.

Another noteworthy observation is that the percentage of retouched pieces in quartz (55%, n = 216) is quite high in comparison to hornfels (32%, n = 123) and dolerite (13%, n = 51). This result is different from previous studies, such as that by Cochrane [41], since this the first technological study to make use of material from all 6 m^2^ of Wadley's excavation of the deep sounding at Sibudu. Previous studies were made on samples from a much reduced area. If we consider all the retouched pieces in the GS layer, quartz pieces comprise more than the half of the total. This retouch representation by rock type seems inversely proportional to the abundance of the three rocks in Sibudu's surroundings (see above). The preferences might be due, on the one hand, to the mechanical properties of these rocks [26] suited to particular tasks at hand or, on the other hand, purely to cultural choice. Although all three rocks (dolerite, hornfels and quartz) are local, dolerite undoubtedly is the most accessible and abundant since it occurs close to the rock shelter. However, the fracture toughness of the dolerite maybe rendered it less suitable to be retouched, shaped and re-sharpened. Instead, quartz was purposely retouched, recycled and valued.

**Figure 14:**
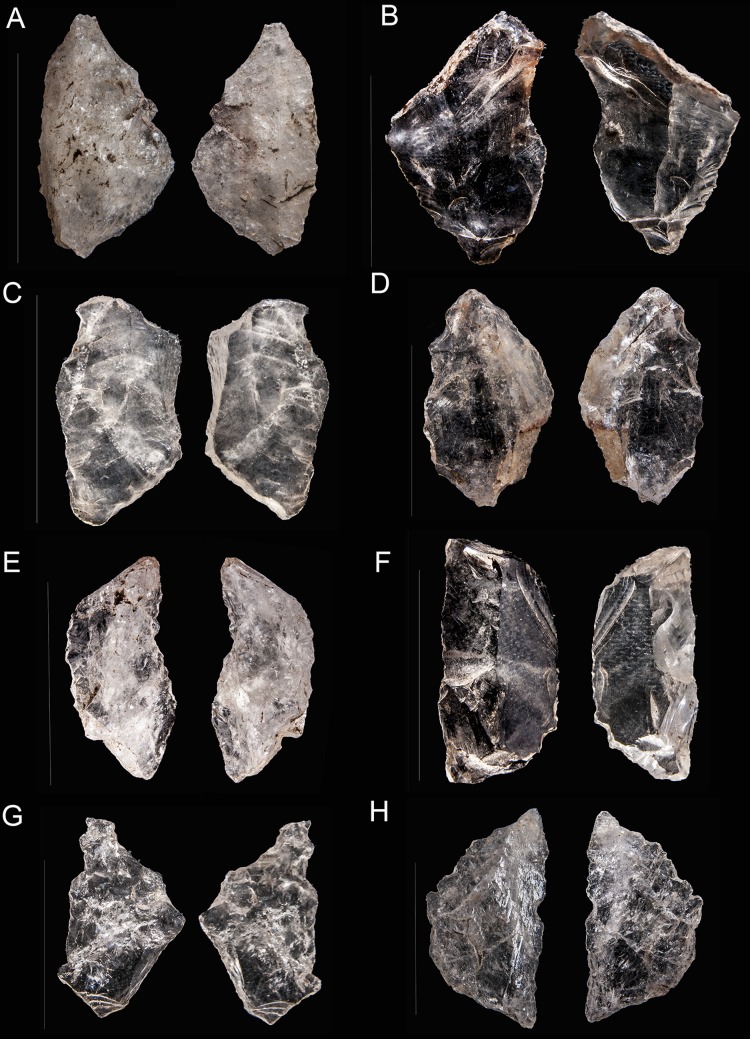
A selection of backed pieces from layer GS. A, D and H, segments. C and F, double truncated pieces. B, E and G, single truncated pieces.

**Figure 15:**
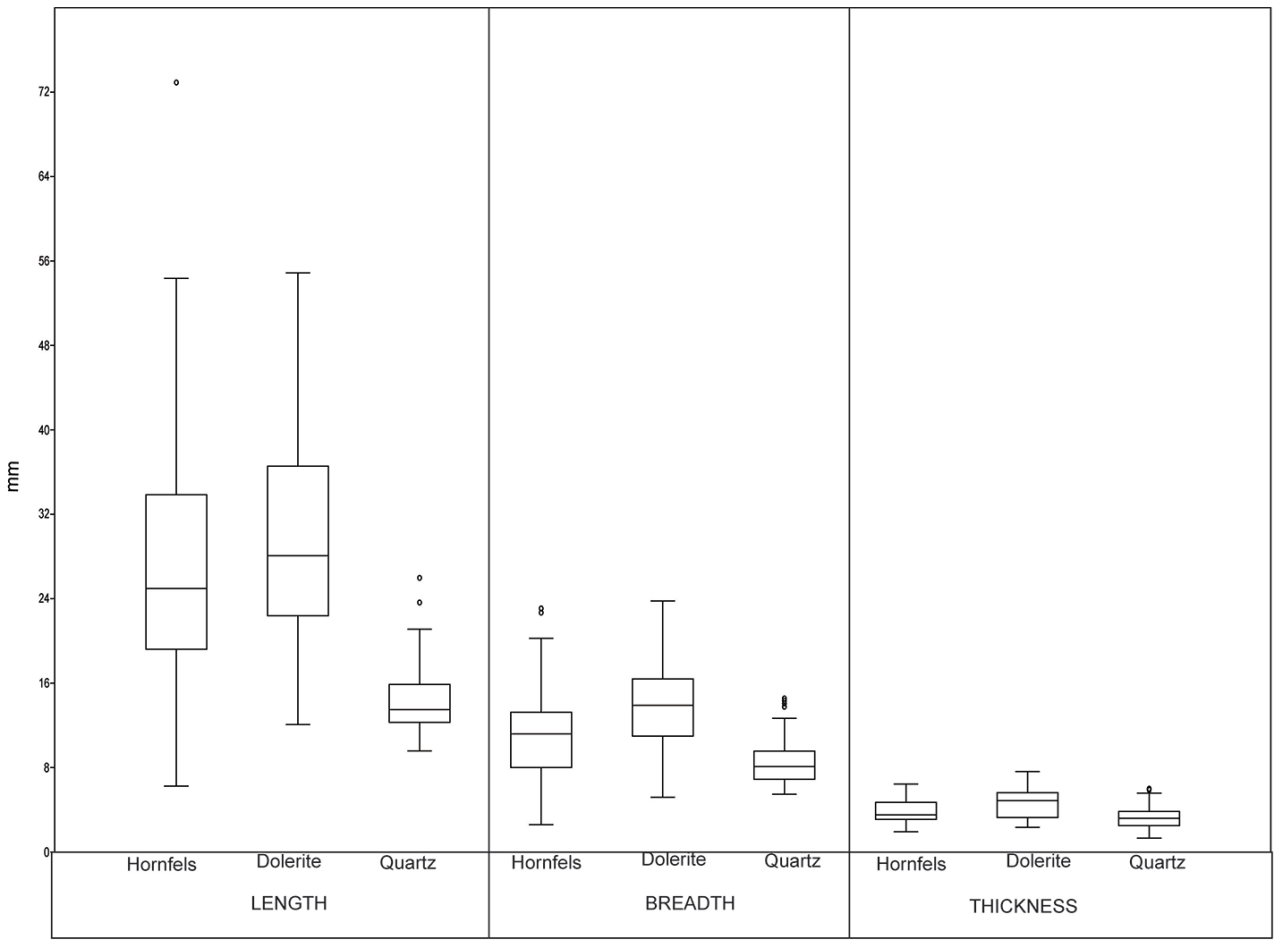
Box-plot of length, breadth and thickness for all backed morphotypes in hornfels, dolerite and quartz from layer GS.

The majority of the retouched pieces in GS are backed tools (such as segments [18.06%] and truncations) (Table 11, Figure 14). It does not seem that there was a preference for lateralization of retouch. Most of the backed morphotypes are made from laminar blanks (56.6%), and probably this percentage should be considered larger, because in many cases it is not possible to recognize which type of blank has been retouched (38.1%). This is because the retouch has eliminated most of the knapping traces. These morphotypes were probably used for hunting purposes [17] [21] [44]. The size range of the quartz backed pieces is particularly small in comparison to that of hornfels and dolerite backed morphotypes (Table 12, Figure 15). This might have functional implications, as has been proposed elsewhere [63].

**Table 11:** Retouched pieces from Sibudu's layer GS in quartz.

	N	%
Borer	5	2,31
End-Scraper	9	4,17
Backed blade/bladelet	3	1,39
Indeterminate backed	16	7,4
Double truncation	3	1,39
Single truncation	16	7,4
Segment	39	18,06
Triangle	5	2,31
Bifacial points (completed pieces and fragments)	37	17,13
Marginal retouched bladelet	10	4,63
Marginal retouched bipolar blank	3	1,39
Retouched flake	14	6,48
Retouched bipolar flake	3	1,39
Double notch	10	4,63
Single notch	32	14,81
Denticulated flake	3	1,39
Denticulated blade	3	1,39
Ind. Denticulated piece	2	0,93
Diverse (piece with some retouch not completed)	3	1,39
Total	216	100

**Table 12:** Univariate data of backed tools in the three main rock types.

	Length			Breadth			Thickness		
	Hornfels	Dolerite	Quartz	Hornfels	Dolerite	Quartz	Hornfels	Dolerite	Quartz
N	43.00	20.00	45.00	77.00	29.00	54.00	71.00	29.00	55.00
Min	6.24	12.08	9.59	2.59	5.18	5.47	1.92	2.33	1.32
Max	72.92	54.87	25.98	23.08	23.79	14.55	6.44	7.63	6.00
Sum	1162.79	611.20	658.79	862.17	406.45	460.50	274.68	135.11	178.52
Mean	27.04	30.56	14.64	11.20	14.02	8.53	3.87	4.66	3.25
Std. error	2.10	2.58	0.54	0.51	0.80	0.31	0.13	0.28	0.14
Variance	190.36	133.25	13.13	20.08	18.73	5.23	1.16	2.34	1.08
Stand. dev	13.80	11.54	3.62	4.48	4.33	2.29	1.07	1.53	1.04
Median	24.97	28.74	13.50	11.20	13.91	8.07	3.53	4.86	3.19
25 prcntil	19.21	22.43	12.25	7.95	10.98	6.85	3.09	3.08	2.50
75 prcntil	33.86	35.70	16.09	13.46	16.85	9.57	4.71	5.76	3.84
Skewness	1.10	0.81	1.28	0.29	0.47	1.12	0.58	0.21	0.62
Kurtosis	1.79	0.26	1.46	0.16	0.14	0.80	−0.48	−0.91	0.38
Geom. mean	23.75	28.58	14.26	10.18	13.35	8.26	3.73	4.41	3.08
Coeff. var	51.02	37.77	24.75	40.02	30.88	26.81	27.78	32.85	32.09

**Figure 16:**
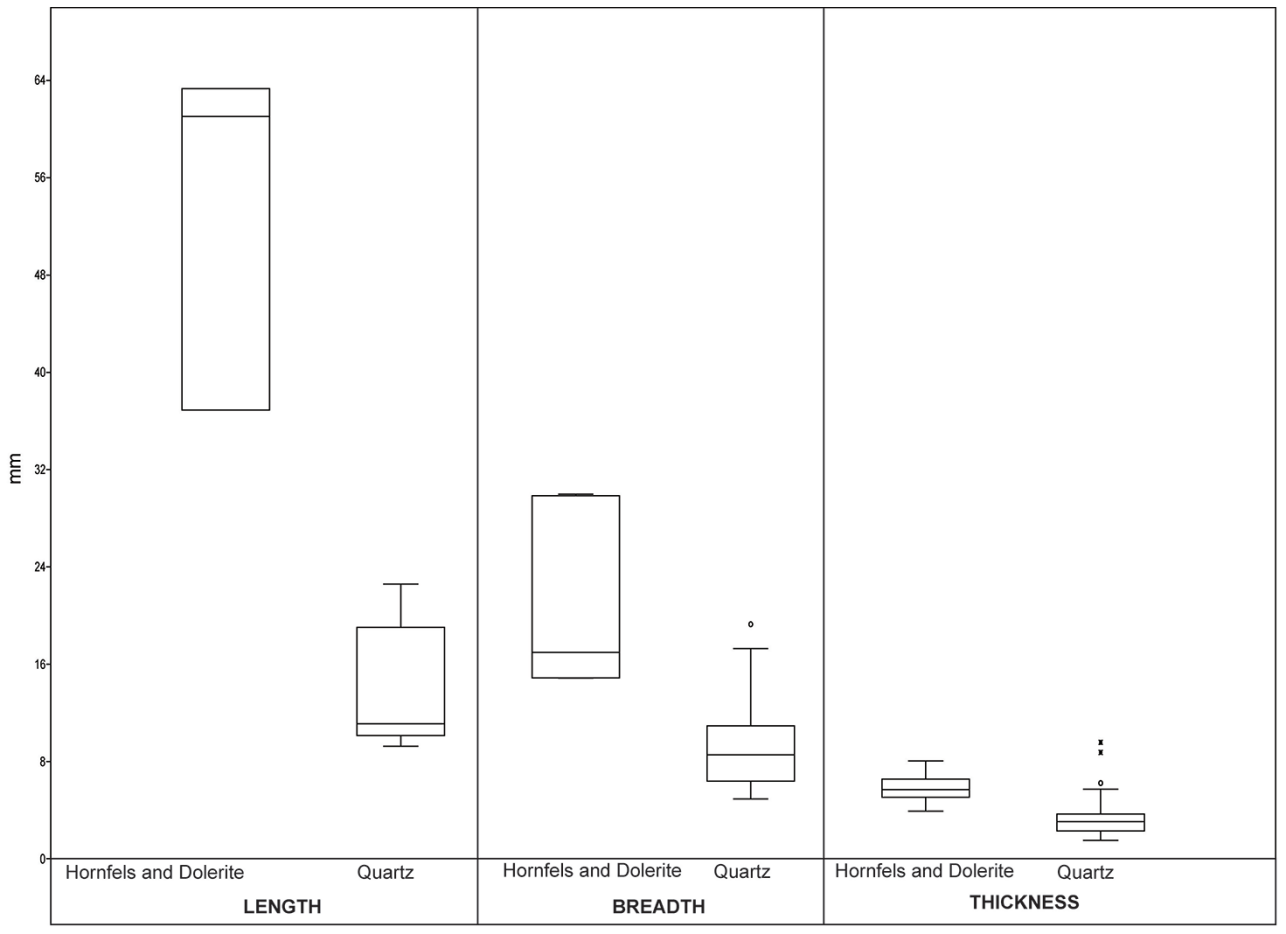
Box-plot of length, breadth and thickness of notched pieces in hornfels,dolerite and quartz.

**Figure 17:**
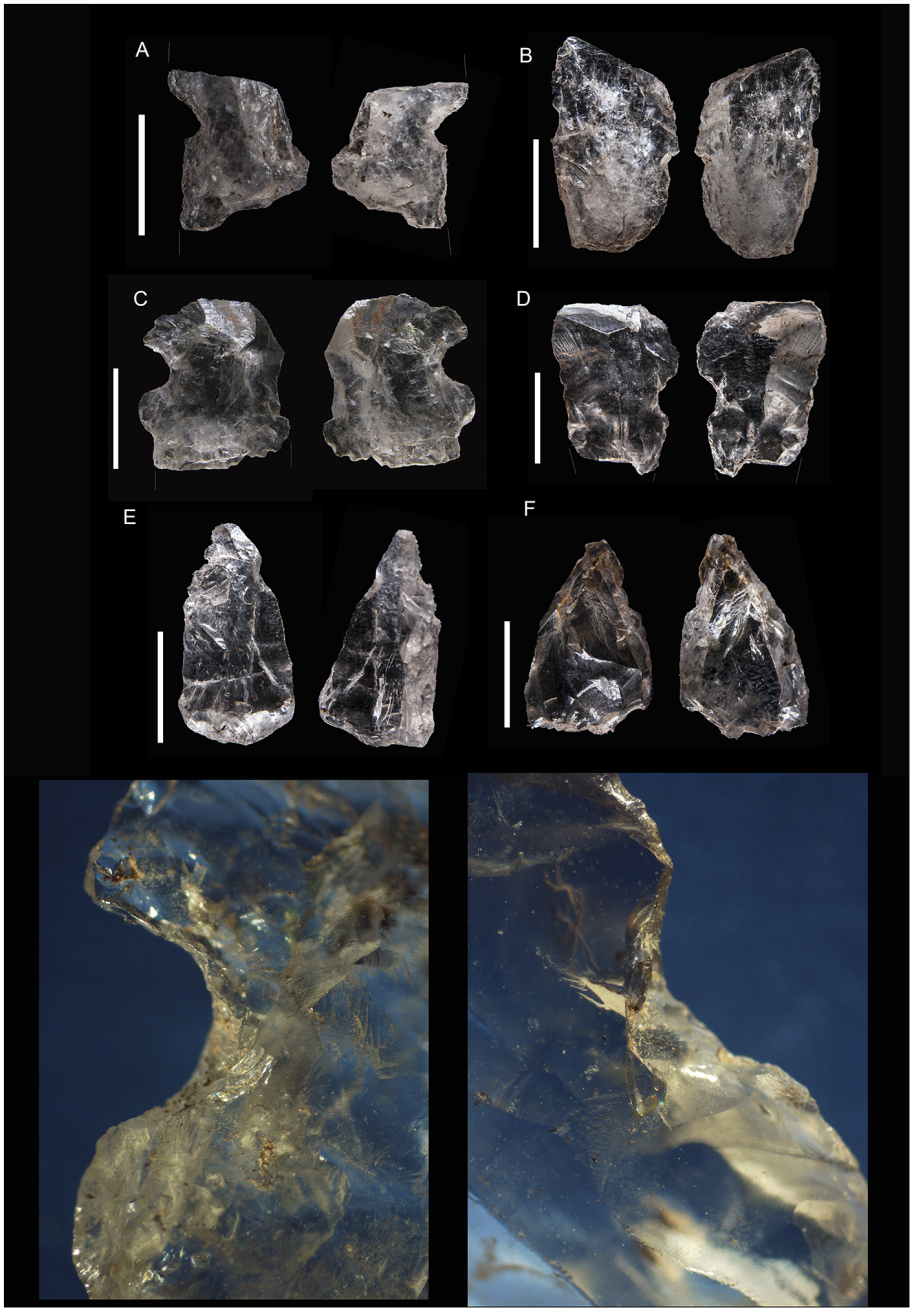
Different examples of quartz notched pieces. A, C and D are single notches. B, E and F are double notches. C′ and D′ are magnifications (x2.5) of the retouched parts of pieces C and D.

The next most common retouched pieces in GS are microlithic notches and double notches (Table 11). These tool types are sometimes smaller than 1 cm and they represent a novelty because the strangulated pieces and notches previously recorded in Howiesons Poort assemblages [58] [59] comprise bigger pieces that may have been used for processing tasks [18]. The retouched notches on the GS quartz tools are particularly small (Table 13, Figure 16 and 17) and have been made on tiny bladelets (57.14% of these morphotypes). Moreover, the notches do not seem to have been the active part of a tool; their small size suggests something like a hafting facility on a bladelet. Clearly this suggestion should be corroborated in the future with functional analysis and experiments; this task exceeds our present aims. The Sibudu tools might be notches from a typological perspective, but their functional dynamic *a priori* seems completely different from that of the strangulated pieces and big notches described in the MSA literature [18].

**Table 13:** Univariate data of notches in the three rock types. The data of hornfels and dolerite have been recorded together.

	Length		Breadth		Thickness	
	Hornfels & Dolerite	Quartz	Hornfels & Dolerite	Quartz	Hornfels & Dolerite	Quartz
N	3.00	17.00	9.00	36.00	10.00	41.00
Min	36.90	9.24	14.86	4.91	3.91	1.51
Max	63.34	22.59	29.98	19.28	8.04	9.57
Sum	161.28	226.51	177.71	332.82	57.93	138.20
Mean	53.76	13.32	19.75	9.25	5.79	3.37
Std. error	8.46	1.10	2.08	0.60	0.42	0.27
Variance	214.52	20.55	38.78	12.96	1.76	3.07
Stand. dev	14.65	4.53	6.23	3.60	1.33	1.75
Median	61.04	11.10	16.98	8.64	5.59	3.05
25 prcntil	36.90	10.13	15.07	6.39	4.82	2.31
75 prcntil	63.34	18.43	26.23	10.91	6.80	3.66
Skewness	−1.68	1.03	1.14	1.13	0.32	2.01
Kurtosis	−2.33	−0.60	−0.42	0.80	−0.45	4.48
Geom. mean	52.25	12.70	18.99	8.65	5.66	3.05
Coeff. var	27.24	34.02	31.54	38.95	22.92	51.96

The third largest group among retouched tools comprises bifacial pieces and a detailed technological analysis of all (not only from GS) the Howiesons Poort bifacial pieces is published elsewhere [30]. The reduction sequence of the quartz bifacial pieces followed a short schema with four potential phases, in which different knapping techniques were involved. The analysis of all the bifacial fragments, preforms and bifacial points has shown that most of the quartz points found in the Howiesons Poort at Sibudu were not finished. This observation contradicts the functional study, which provides evidence of use on unfinished pieces even where this was unexpected from a technological point of view. Two of the bifacial points display use-wear, striations, macroscopic fractures and residues that link them most plausibly with hunting tasks [30].

It must be noted that, when combined, backed pieces, small notches and bifacial pieces reach almost 75% of the retouched pieces in quartz. In comparison, the percentage of quartz processing tools (such as end-scrapers, borers and denticulates) is quite low, perhaps because hornfels and dolerite were preferred for these tools. It is possible that retouched flakes belong within the generic group of processing tools, but further investigation must be carried out before this can be confirmed.

## Discussion

We first summarise what we have learnt about Sibudu's layer GS quartz technology. Quartz river pebbles were transported to the site, and some of them were knapped in a discoidal or multifacial way for the production of flake blanks for bifacial points. Other pebbles were directly knapped to produce laminar blanks (or elongated products). Finally, many of the quartz waste-remains, and probably a lot of freehand cores, were recycled for bipolar knapping in order to obtain small bipolar blanks. Quartz was exploited extensively in GS at Sibudu, and the different knapping methods involved appear to have been destined for different tool sets. After production it seems that many blanks were used without any kind of retouch, but suitable flakes were transformed into bifacial pieces, while backed pieces (mostly segments) were made from some of the bladelets. Other bladelets had one or two small notches delicately retouched on their laterals; the function of these tools is still an enigma, but we plan use-trace analysis and experiments in the future. The selection of quartz for knapping is noteworthy for two reasons: first, quartz is difficult to knap because of its faults and fracture planes (especially for freehand production); secondly, the small size of the nodules adds an additional level of difficulty to the knapping strategies involved.

What is particularly conspicuous in this quartz reduction sequence is the recycling strategy of bipolar knapping, and the extremely small size of some of the desirable products, such as the bladelets, the bipolar blanks, the segments and the small notches.

The choice of rock types in Sibudu does not demonstrate long-distance sourcing strategies; all the rocks used at Sibudu are essentially local or come from a maximum of 20 km from the site [63]. Rose Cottage Cave is another striking example of local rock collection; opalines were used throughout the long sequence from the pre-Howiesons Poort to the LSA [64] [22]. Several other sites, such as Nelson Bay Cave and Klasies River, have an increased use of quartz in their Howiesons Poort assemblages. Quartz was also valued at Umhlatuzana (not far from Sibudu in KwaZulu-Natal) [65]. Some sites reveal a preference in the Howiesons Poort for the production of blade/bladelets and backed geometric pieces from fine-grained rocks as well as quartz [16] [21] [34] [59] [66]. As we explained earlier, crystalline quartz is, in the geological sense, a mineral that is not granular; nonetheless it has been listed as ‘fine grained’ in several southern African studies [56] [67]. Only impure varieties of quartz contain inclusions such as sand, clay or iron oxide [45].

Bipolar knapping is a straightforward method and its occasional appearance has even been documented in Early Stone Age assemblages [68]. In this early stage of lithic production, bipolar knapping may have been an expedient technique, but this seems not to be the case when it was used consistently and with regularity in the MSA. The two main reasons for its use are: first, bipolar knapping makes it possible to exploit small nodules that would be impossible to process by freehand knapping [50] [62], so bipolar knapping provides potential for a recycling strategy after initial freehand percussion. Secondly, bipolar knapping implies an explosion in numbers of small elements, in other words, it introduces a microlithic strategy for the production of small blanks. Therefore, as Callahan [50] pointed out, bipolar knapping is a simple knapping method, but not simplistic, and its application involves a clever economy of rock management.

On occasion, scaled pieces and bipolar cores are conflated in MSA literature. We highlight this debate because such pieces have now and then been defined loosely, including in Howiesons Poort assemblages. The common confusion between scaled pieces and bipolar cores was pointed out years ago in other geographic areas, and in other periods [69], for example, in Paleo-Indian archaeology [70], Early Upper Palaeolithic contexts in Western Europe [71] and lately in Early Stone Age sites [53]. In southern African studies the categories of splintered piece, *pièces esquillées, outils écaillés*, and core reduced pieces are generally used in an ambiguous way or inter-changeably. However, the terms are typological constructs; they lack technological or functional meaning and they have been interpreted in different contexts either as bipolar cores or as intermediary tools to wedge hard materials. In South Africa we cite the pioneering work of Van Riet Lowe [72], as one of the first archaeologists to recognize this type of knapping and, much later, Barham's experimental work [73], which contributed significantly to the identification of bipolar knapping in African contexts. Recently Igreja and Porraz [18] interpreted splintered pieces as intermediate tools in the Howiesons Poort layers of Diepkloof. Moreover, in her pioneering work, based on use-trace analyses of a few of Sibudu's bipolar products from the Howiesons Poort (including two GS pieces), Langejans [74] cautiously concluded that they were used for animal related activities. However, she does allow that the animal residues might have accumulated from bone hammer percussion or cores later used as tools. There has been a suggestion, though, that bipolar cores may have been wrapped while they were being hammered [75] and this may have resulted in residues from leather. We believe that the combination of a technological and a use-wear macroscopic approach to these types of pieces is the most suitable procedure [55] [56].

In this paper we interpret the Sibudu splintered pieces as bipolar cores because of our attribute analysis (based on previous studies of quartz knapping) and after discovering and describing the quartz blank production. A technological justification is always required to defend either an interpretation of the splintered pieces as cores, or as tools.

Extensive bipolar knapping is accepted as part of LSA technology [1] [4] [8] [76] [77]. Indeed, as we mentioned in the beginning, it has sometimes been used as a technological marker to indicate the arrival of the LSA (merely because of its appearance or because it has a high frequency in LSA contexts), whereas bipolar knapping in the MSA was treated ambiguously. The demonstration that quartz bipolar knapping is a substantial component of Howiesons Poort technology in layer GS at Sibudu dismantles the notion that this strategy is especially associated with the LSA or can be used as a marker for recognising the appearance of the LSA.

Quartz bladelet technology is prolific in layer GS and, as has been demonstrated, the production was focussed on obtaining tiny bladelets to be used without retouch, or to be converted into small backed pieces or other morphotypes (such as small single or double notches).

Kuhn and Elston's [78] ideas for differentiating microlithic industries were designed for other geographical areas and time spans, but they seem useful for application to the African situation. In the first place, microlithic strategies are related to the proliferation of bladelet or microblade core technology for the production of small, elongated blanks. Secondly, when such blanks are retouched, the modification generally takes the form of backed retouch. Another characteristic is standardization of size or shape, or both. Wadley and Mohapi [63] demonstrated that the quartz segments in Sibudu's Howiesons Poort have highly standardized shapes. In order to assign the term ‘microlithic strategy’, microlithic pieces need to make up a numerically dominant component of lithic assemblages. Nonetheless, for researchers such as Flenniken [79], the massive production of small stone artefacts, with or without retouch, and whether or not they are standardized, is enough to talk about a microlithic strategy. He proposed a microlithic strategy for the Hoko River Site, using as attributes bipolar knapping and composite tools with wooden hafts. In the southern African context we point out that the Holocene Wilton Industry contains microlithic strategies that are not always related to bladelet/microblade production for making small, elongated blanks. Furthermore, the Robberg Industry demonstrates a very clear situation in which such small, elongated blanks are almost never modified further, by backing or any other kind of retouch [75] [80] [81]. In GS, at Sibudu, the considerable bipolar production in the Howiesons Poort (demonstrated by the large representation of bipolar cores and the high frequency of used bipolar blanks) fits into Flenniken's [79] description. Furthermore, the backed quartz pieces from GS fall completely within Kuhn and Elston's [78] definition of microliths. In addition, it seems that, on a broader scale, quartz knapping in GS is part of a microlithic strategy. In this regard Table 14 compares the sizes of quartz backed tools and segments from GS with those made on other rock types within the same layer, and also from other southern African MSA and LSA sites. Quartz is not particularly popular in most of Sibudu's MSA sequence so its use in the Howiesons Poort seems deliberate, not expedient.

**Table 14:** Comparison of mean lengths and coefficients of variation (CV) of segments and/or backed tools from selected Howiesons Poort and LSA sites.

	Site	Mean Length (mm)	CV	N	Reference
**Howiesons Poort**	Sibudu dolerite all backed tools (Grey Sand)	30.56	37.77	20	See text this paper
	Sibudu hornfels all backed tools (Grey Sand)	27.04	51.02	43	
	Sibudu quartz all backed tools (Grey Sand)	14.63	24.75	45	
	Sibudu quartz segments (Grey Sand)	14.61	23.72	30	
	Rose Cottage Cave all backed tools	27.3	27	39	Soriano et al., 2007
	Klasies River. Singer & Wymer collection (Cave 1A) All materials	36.6	25.8	630	Data facilitated by S. Wurz, personal comunication
	Klasies River. Singer & Wymer collection (Cave 1A) Milky quartz	27.4	22.7	27	
	Klasies River. Singer & Wymer collection (Cave 1A) Milky and glassy quartz	27.2	21.4	33	
	Klasies River. Deacon collection (Cave 1A). All materials	35.1	27.7	28	
	Klasies River. Deacon collection (Cave 2). All materials	36.6	29	58	
**Later Stone Age examples**	Nelson Bay Cave segments	46.1	16	45	Wurz 2000
	Montagu Cave segments	29.9	23	37	
	Border Cave backed tools	47.7	-	16	
	Uniondale backed tools	17.2	19	178	
	Mumba backed tools	34.2	27	29	
	Melkhoutboom backed tools	11.96	24	101	
	Wilton all layers backed tools	15.4	26	54	
	Wilton, layer 3F backed tools	16.8	6	27	
	Jubilee quartz backed tools	9.3	28	15	Delagnes et al. 2006
	Jubilee segments	10.03	24	41	

Note that quartz backed tools and segments from GS fall in the same distribution that some of the LSA considered as microlithic (see Wilton example) examples.

The quartz technology of Sibudu's layer GS has several interesting implications for debates about the MSA and LSA and the transformation from the one to the other. First, quartz technology provides an example of variability within Howiesons Poort assemblages, and technological variability in the MSA has already been highlighted by others [34]. Having said this, it must be pointed out that no prior technological studies focus exclusively on quartz in Howiesons Poort contexts. The use of bipolar knapping as a recurrent recycling and microlithic strategy for quartz has not yet been recorded in any publications of other Howiesons Poort sites, but see [82]. Perhaps this is due to regional variation, but perhaps other analysts chose not to study quartz pieces, which are difficult to work with. Moreover, the discovery of these strategies in the Howiesons Poort refutes an interpretation of technology in the MSA as cumulative. The appearance and disappearance of technological variants should be seen then as a result of people seeking specific solutions to problems in the past. The technological decisions were mediated by functional, cultural and symbolic choices, and they were not part of a technologically complex evolutionary trajectory, because the record indicates otherwise [35]. Therefore, bipolar knapping should not be contemplated as a milestone of ‘modern behaviour’, but simply as a techno-functional strategy. In LSA industries it is accepted that there is great variability amongst microlithic and non-microlithic industrial variants. Indeed, some LSA industries are considered microlithic without having either backed retouched tools, or a fully developed bladelet technology [4] [77]. As we have tried to show here, it appears that such intermittent variability is also typical of the MSA.
